# Effect of different velocity loss thresholds during a resistance training program on jump and sprint performances in trained female athletes

**DOI:** 10.1371/journal.pone.0347298

**Published:** 2026-04-13

**Authors:** Hiroki Kambara, Kazuhiro Sakamoto, Yuya Watanabe, Mitsuo Neya

**Affiliations:** 1 Faculty of Sport Study, Biwako Seikei Sport College, Otsu-shi, Shiga, Japan; 2 TOYOTA VERBLITZ, Toyota Rugby Team, Toyota-shi, Aichi, Japan; Instituto Politecnico de Santarem Escola Superior de Desporto de Rio Maior, PORTUGAL

## Abstract

This study aimed to explore the effects of velocity-based resistance training (VBT) using different velocity loss (VL) thresholds on jump and sprint performance in trained female athletes. Fifteen college-level female basketball players completed an 8-week VBT program (2 sessions/week), involving parallel back squats performed at a target mean propulsive velocity of 0.7 m/s. Participants were randomly assigned to two groups: VL10% (n = 8) and VL20% (n = 7), where training sets were terminated when the target velocity-loss threshold was exceeded for the second time within the same set. Performance tests, including one-repetition maximum (1RM), squat jump (SJ), countermovement jump (CMJ), and 20-m sprint (SP20) with split times recorded at 5 m (SP5) and 10 m (SP10), were conducted pre- and post-intervention. The VL10% group showed significant improvements in SJ (p = 0.048, d = 1.30), SP10 (p = 0.004, d = 0.62), SP20 (p = 0.002, d = 0.67), and 1RM (p = 0.002, d = 0.29). The VL20% group also showed improvements in SP10 (p = 0.004, d = 0.42), SP20 (p = 0.002, d = 0.56), and 1RM (p = 0.002, d = 0.62), although SJ did not significantly improve. Despite no significant interaction effects, effect sizes suggest possible differences that require verification in adequately powered trials. VBT using low VL thresholds may be useful for maintaining movement velocity with lower training volume; however, between-group differences were not statistically significant and CMJ did not show clear improvement. Larger-scale studies are needed to confirm these trends.

## Introduction

Conventional resistance training has been recognized as percent-based training (PBT), in which training weight is determined as a percentage of one repetition maximum (%1RM) and the amount is defined by the number of repetitions and sets [1]. However, it does not consider the physiological and psychological stresses that affect daily training [2,3].

Velocity based training (VBT) has been attracting attention in recent years. VBT is a method of training while motivating athletes by monitoring their velocity in real time after each repetition and providing visual and verbal feedback such as velocity and power [4,5]. VBT requires that the concentric phase of the exercise is performed with maximum intended velocity, and the velocity of each individual repetition is measured [6]. VBT prescribes exercise load based on target movement velocity, while velocity loss (VL) thresholds are used to monitor fatigue and determine set termination [7]. Previous studies have examined the optimal magnitude of VL to improve performance, as different VL thresholds influence the total training volume performed during a session. These thresholds do not determine external load, but indirectly influence training volume by regulating set termination. Pareja-Blanco et al. [8] examined the effects of different velocity loss thresholds on countermovement jump (CMJ) and sprint performance in trained male participants by comparing VL15% and VL30% training protocols. Despite a substantially greater number of repetitions performed in the VL30% condition, no significant differences were observed in CMJ or sprint performance between the pre- and post-training periods. Similarly, Rodriguez-Rosell et al. [9] compared the effects of VL10%, VL30%, and VL45% training on jump and sprint performance. Their results also showed no significant pre- to post-training differences in these performance measures, despite large differences in training volume across velocity loss conditions. In line with these findings, Galiano et al. [10] investigated the effects of low (VL5%) and moderate (VL20%) velocity loss thresholds in a relatively large sample of physically active men and reported comparable improvements in strength, jump, and sprint performance between conditions, despite substantially lower training volume in the VL5% group. These results further support the efficiency of low velocity loss training for inducing neuromuscular adaptations in male populations. However, it remains unclear whether such findings can be directly extrapolated to female athletes, who may exhibit distinct neuromuscular and fatigue-related characteristics. Recent literature has also highlighted the growing role of biomechanics in optimizing athlete performance and informing monitoring-based training decisions, providing broader context for the applied use of VBT in sport settings [11]. Taken together, these findings suggest that training with lower velocity loss thresholds (e.g., VL5%–10%) may be equally or more effective for improving jump and short sprint performance compared with training using higher velocity loss thresholds (≥20%), while requiring a substantially lower number of repetitions. In addition, Pareja-Blanco et al. [12] reported that only one repetition per set (VL0%) is insufficient to maximize strength gains, while a high VL threshold (VL40%) does not lead to further strength gains. Therefore, extremely low VL (e.g., VL0%) may be insufficient to maximize performance improvements. Pareja-Blanco et al. [8,12] demonstrated that relatively higher VL (20% or more) is more effective for muscle hypertrophy and strength gains. These studies have shown that appropriate VL should be set depending on the training purpose. Recent systematic reviews and meta-analyses published within the last few years have highlighted velocity loss thresholds as a key variable for modulating neuromuscular adaptations during velocity-based resistance training, while also emphasizing the limited number of controlled intervention studies in female athletes [4,5]. Previous studies have demonstrated sex-related differences in muscle morphology, strength, and neuromuscular function [13,14]. However, evidence from VBT intervention studies in female athletes remains limited [4]. Rissanen et al. [15] compared countermovement jump (CMJ) height in men and women training with VL20% and VL40% protocols and observed significant increases in CMJ height, with no differences between velocity-loss groups. In addition, they reported that women performed a greater number of repetitions than men.

Although between-group differences were not statistically significant, the authors noted larger magnitudes of improvement in women following the 40% velocity-loss condition, particularly for bench press outcomes, suggesting that women may require greater velocity loss to maximize certain adaptations.

Gender differences in muscle properties may be associated with this result. Simoneau et al. [16] reported that the percentage of muscle fiber type I (slow muscle fibers) was higher in female than in male. In addition, Hakkinen et al. [17] found that female had less neuromuscular fatigue than male, and acute recovery from fatigue was faster in female than in male [18,19]. Furthermore, they reported that blood lactate levels were lower in female than in male [20].

VBT performed at lower than VL20% has been associated with improvements in vertical jump performance, primarily assessed by countermovement jump (CMJ), and sprint performance in male. While these findings are well-documented in male populations, research on female athletes remains limited. Gender-based physiological differences—including higher type I muscle fiber distribution, greater fatigue resistance, and faster recovery from high-intensity efforts—suggest that optimal VL prescriptions may differ between men and women. Nonetheless, few studies have investigated how VL thresholds influence performance adaptations in female athletes, and even fewer have considered the implications in applied sport settings such as collegiate basketball. Recent systematic reviews and meta-analyses have emphasized that velocity loss thresholds are a key variable for modulating adaptations during velocity-based resistance training [3,4]. However, these reviews also highlight the limited number of controlled intervention studies in female athletes, particularly examining task-specific outcomes such as jumping and sprinting performance.

Given the growing implementation of VBT in athletic programs and the lack of sex-specific evidence, further investigation is warranted. Collectively, these studies suggest that lower velocity loss (VL) thresholds (≤10%) tend to emphasize velocity preservation and high-velocity neuromuscular adaptations, whereas moderate VL thresholds (≈20%) are more strongly associated with greater volume accumulation and strength-oriented adaptations. Therefore, we selected VL thresholds of 10% and 20% to represent two distinct, methodologically supported training strategies. This study aimed to explore the effects of VBT using two commonly applied VL thresholds (10% and 20%) on jump and sprint performance in trained female basketball players. Based on previous findings indicating that lower VL thresholds favor the preservation of movement velocity and high-velocity neuromuscular adaptations, we hypothesized that the VL10% group would exhibit greater improvements in jump and sprint performance than the VL20% group [4,21].

The findings are intended to serve as foundational data for future large-scale trials and to inform practical training strategies for female athletes.

## Methods

### Experimental design

Study design: This study was a two-arm, parallel-group intervention trial (VL10% vs VL20%) with pre–post performance assessments. Performance tests were conducted in the following order: (1) SJ and CMJ, (2) 20-m sprint (SP5, SP10, SP20), and (3) 1RM back squat, with standardized rest intervals as described below.

**Fig 1:**
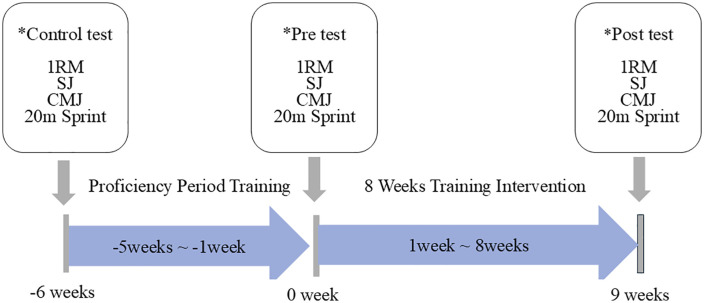
Overall experimental study design timeline. 20-m sprint (5 m, 10 m, 20 **m)**. 1RM indicates 1-repetition maximum; SJ, squat jump; CMJ, counter movement jump.

This intervention study was designed to examine the effects of VBT using different VL on jump and sprint performances in female athletes. Participants trained twice a week (48−72 h apart) over 8-week (Week-1 to Week-8) period for the total of 16 sessions. Both groups trained with a target velocity of 0.7 m/s (70% 1RM) for each squat (SQ) session, but there was a difference in the maximum %VL reached in each exercise set (10% vs. 20%). The set was terminated when the target VL threshold was exceeded for the second time within the same set. Using velocity loss to terminate sets is a widely used approach in velocity-based training to regulate neuromuscular fatigue and standardize training stimulus across sessions [7,21]. Participants were tested three times during the period: proficiency period, before training (Week 0, Pre), after intervention program (Week 8, Post) [22]. The order of testing (jump tests, sprint tests, and 1RM) was selected to minimize fatigue effects, following general testing recommendations. In addition, this order was consistent with the team’s regular testing routine, ensuring familiarity for the participants. A standardized rest period of 30 min was provided between the 1RM test and the subsequent performance assessments, while a 3-min rest interval was allowed between each of the remaining assessments (Fig 1). The assessment was conducted one week before the training period (Pre) and one week after the training period (Post). A single National Strength and Conditioning Association (NSCA)-certified strength and conditioning specialist supervised all of the assessments and training sessions.

### Participants

Participants were collegiate sub-elite female basketball players. The intervention was conducted during the pre-season period, during which the team trained six times per week. Playing positions included point guard, shooting guard, small forward, power forward, and center. All participants had at least 6 years of basketball experience. Although 28 [VL10%: n = 14, 20.3 (1.3) years, 161.4 (5.8) cm, 58.1 (6.7) kg, VL20%: n = 14, 20.1 (0.8) years, 162.2 (6.6) cm, 58.4 (9.5) kg] participants were initially recruited, only 15 (VL10: n = 8; VL20: n = 7) completed the 8-week training intervention. Reasons for dropout unrelated to the study protocol included, for example, acute injury, illness and scheduling conflicts. Despite the limited sample size, this investigation provides provisional results into VBT responses among female athletes. Before the assessments and training, all players were fully informed by written document and verbally about the experimental procedures, and the purpose, the potential risks, and the benefits associated with this study. Written informed consent was obtained from each subject before measurements commenced. The study was approved by Research Ethics Review Committee of Biwako Seikei Sport College (IRB2022−7). This study began on January 30, 2023, and ended on September 30, 2023 (from the recruitment of research participants to the collection of all data).

### Intervention program

The main exercise in the training program was the free-weight parallel back squat. In addition, dynamic stretching and upper-body exercises were included, and supplementary trunk exercises targeting the abdominal muscles were performed at the end of each training session. Participants were instructed to perform the eccentric phase until reaching a standardized parallel squat depth, defined as the position at which the hamstrings were approximately parallel to the floor, followed by a concentric phase performed with maximal intended velocity. To ensure consistent range of motion across repetitions, a cord was placed at the individual parallel depth during training sessions, and participants initiated the concentric phase of each repetition when the gluteal region contacted the cord; this procedure was implemented during the familiarization period to allow participants to consistently reproduce their individual parallel squat depth throughout the intervention. Mean propulsive velocity (MPV) of each testing load was recorded using a linear position transducer (GymAware Power Tool; Kinetic Performance Technologies, Australia). We acknowledge recent calls for standardization in the algorithmic assessment of sensor technologies used in sport performance monitoring [23].

The exercise load was determined by the weight that could be lifted at a velocity of 0.7 m/s. Five repetitions were performed at 20 kg and two repetitions at the load used in the training session as warm-up before beginning the training session. The target MPV of 0.70 m/s was selected to establish a stable and reliable reference point for daily load prescription while minimizing excessive pre-test fatigue. Submaximal velocities around this range have been reported to provide a reliable load–velocity relationship for estimating relative loading in the back squat [4], and a similar reference velocity has been used as a practical criterion in previous work examining velocity loss during resistance training [18]. In addition, 0.7 m/s corresponds approximately to ~70% 1RM in the back squat, representing a practical loading range that can provide sufficient mechanical stimulus for both hypertrophic- and strength-related adaptations. Velocity was measured during the two repetitions, and if the average velocity differed by more than 0.06 m/s above or below the target velocity, the load was increased or decreased by 5% of 1RM; if the difference exceeded 0.12 m/s, the load was increased or decreased by 10% of 1RM, consistent with previously described velocity-based load adjustment procedures [2,7]. The inter-set rest interval was 2 min in all sessions. Strong verbal encouragement was used in all training sessions to perform the concentric phases with the maximal intended velocity. Each participant performed an individual number of repetitions in each training session based on their velocity-loss allocation. Velocity loss was monitored on a repetition-by-repetition basis. The set was terminated when the target velocity loss threshold was exceeded for the second time within the same set. This approach was adopted to avoid terminating the set due to a single potentially spurious repetition, as velocity reductions observed in the first occurrence may not necessarily reflect true neuromuscular fatigue despite the familiarization period.

Each training session consisted of a standardized warm-up, a main resistance exercise, and supplementary exercises. The warm-up included dynamic stretching targeting the hip joint musculature, followed by squat-specific warm-up sets (five repetitions at 20 kg and two repetitions at the training load). The main exercise was the free-weight parallel back squat performed under VBT conditions as described above. After completion of the main exercise, participants performed supplementary upper-body and trunk exercises, including abdominal exercises, which were standardized across sessions. This structured training sequence was applied consistently throughout the intervention period. Details regarding warm-up procedures performed prior to performance testing are described separately in the corresponding assessment sections.

### Performance tests

#### 1RM test.

The same method as in the intervention program was used to measure 1RM. In addition, a “load-velocity profile (LVP)” was created to clarify the relationship between weight and velocity used in the intervention program [2,24]. Subjects were measured from light weight to maximum weight along with velocity to create LVP. The 1RM was defined as the maximum weight that a participant was able to lift once in a full range motion. The 1RM was determined as follows. The participants performed a warm-up consisting of 8 and 6 SQ repetitions with 20 and 30 kg loads, respectively. After the warm-up, the participants attempted to measure their 1RM. The initial load for the 1RM measurement was 30 kg. The load was then increased by 10 kg increments until the MPV of the attempt was less than 0.5 m/s. After that, for better adjustment, measurements were taken with small increments of 2.5 kg. Repeated measurements were taken three times at light loads (>1.00 m/s), twice at medium loads (1.00 m/s > 0.80 m/s), and only once at the heaviest load (<0.80 m/s). A 2-min recovery period was allowed between trials. The load at the point when the participants could no longer lift the load or the lifting velocity become less than 0.3 m/s was defined as 1RM [12,25]. The 1RM testing and warm-up procedures were conducted following commonly used recommendations for maximal strength testing, and recent work has further discussed practical 1RM testing/prediction considerations in squat-based assessments [26].

#### 20m Sprint test.

Before the sprint tests, participants completed a standardized warm-up consisting of four dynamic stretching exercises targeting the hip region, followed by squat exercises performed for 8 and 6 repetitions with external loads of 20 kg and 30 kg, respectively. This sprint-specific warm-up approach is consistent with previous studies reporting performance benefits of dynamic warm-up strategies prior to high-intensity running tasks [27]. The participants carried out two maximal 20-m sprints. Sprint times were measured using photocells (Witty: Microgate, Bolzano, Italy). All sprint tests were performed on an indoor hardwood court. Photocell timing gates were set at 0, 5, 10, and 20 m so that the times to cover the 0–5 m (SP5), 0 – to 10 m (SP10), and 0–20 m (SP20) distances could be determined. A standing start with the lead-off foot placed 0.5-m behind the first timing gate was used. The better time of 2 attempts was used for analysis. Times recorded were converted to velocity (m/s) based on distance/time.

The coefficients of variation (CV) for test-retest reliability for SP5, SP10, and SP20 were 0.78, 0.92, and 0.95, respectively. The intraclass correlation coefficients (ICC) were 0.80 (95% confidence interval, CI: 0.51–0.93) for SP5, 0.92 (95% CI:0.79–0.97) for SP10, and 0.95(95% CI:0.86–0.98) for SP20.

#### Jump tests.

Jump test was measured using an infrared timing system (Optojump Next: Microgate, Bolzano, Italy). Before the SJ and CMJ assessments, participants completed their team’s regular warm-up routine prior to the start of testing.

SJ: In an upright posture, the knee angle was set at 90°, which was verified using a goniometer prior to movement initiation, and a maximum vertical jump was performed without countermovement. Two trials were completed with a 20 s rest between trials, and the higher value was used for analysis. The CV was 0.93 and the ICC was 0.94 (95% CI: 0.83–0.98).

CMJ: A fast downward movement was immediately followed by a fast upward vertical movement performed as high as possible in a single continuous sequence. No specific knee joint angle or countermovement depth was prescribed, and participants performed the jump using a self-selected countermovement depth. Two trials were completed with a 20-s rest between trials, and the best value was used for analysis. The CV was 0.98 and the ICC was 0.98 (95% CI: 0.95–0.99).

All assessments were conducted by the same examiners, who were responsible for test preparation, instruction, and data collection throughout the study. Prior to the intervention, participants had completed at least one full battery of the same tests as part of their regular team testing routine, ensuring familiarity with all assessment procedures.

### Sample size

This study was designed as a pilot, exploratory trial; therefore, a formal a priori sample size calculation was not performed prior to participant recruitment.

To inform future confirmatory research, an a priori power analysis was conducted using G*Power (version 3.1.9.7) for a 2 (group) × 2 (time) repeated-measures ANOVA (within–between interaction). Assuming a medium effect size (f = 0.25), an alpha level of 0.05, statistical power of 0.80, correlation among repeated measures r = 0.50, and a nonsphericity correction of ε = 1.0, the required total sample size was estimated to be 34 participants (17 per group). The initial recruitment target for the present study was 28 participants (14 per group), which approached this estimate and was considered reasonable for a pilot study in trained female athletes. However, participant attrition due to injury resulted in a smaller final analytical sample.

### Statistical analysis

The normality of each outcome variable (pre and post) was assessed using the Shapiro–Wilk test, and homogeneity of variance between groups was assessed using Levene’s test. For variables meeting parametric assumptions (1RM, SP10, and SP20), a two-way mixed ANOVA (group × time) was applied, and Bonferroni-adjusted post hoc comparisons were used when appropriate. For variables not meeting parametric assumptions (SJ, CMJ, and SP5), within-group pre–post changes were analyzed using the Wilcoxon signed-rank test, and between-group differences in change scores (Δ = post − pre) were analyzed using the Mann–Whitney U test. Effect sizes (Cohen’s d) were calculated to aid interpretation. The values for assessing magnitudes of ES were 0.20, 0.60, 1.20 and 2.00 for small, moderate, large, and very large, respectively [25]. All analyses were performed using SPSS (version 28.0; IBM Corp., Armonk, NY, USA), with the significance level set at p < 0.05.

## Results

Of 28 participants identified, 15 (VL10: n = 8; VL20: n = 7) were enrolled. Thirteen subjects dropped out during the experimental period for reasons unrelated to this study protocol. Based on the results of Shapiro-Wilk test and Levene’s test, variables of 1RM, SP10, and SP20 were analyzed using parametric procedures. The other variables were analyzed using non-parametric procedures.

The VBT status in this study throughout the intervention period was summarized in Table 1. The number of total repetitions during the intervention period and mean repetitions per session period were smaller in the VL10% group than in the VL20% group. The number of repetitions per session in the VL10% group was approximately 52% of that performed by the VL20% group, indicating that participants in the VL10% group completed about half the number of repetitions per session compared with the VL20% group. The fastest lifting velocity per session remained close to the target velocity in both groups. The mean VL was 10.9% in the VL10% group and 22.0% in the VL20% group. We interpreted that both groups achieved their respective target VL.

**Table 1:** Descriptive characteristics of an 8-week velocity-based back squat training program conducted by two experimental groups.

	Total Repetitions	Mean Repetitions/ Session	Fastest MPV (m/s)	Slowest MPV (m/s)	Mean VL (%)
VL10%	2260*	17.8 (5.20)*	0.71 (0.03)	0.63 (0.03)	10.93 (1.79)
VL20%	3859*	34.5 (6.73)*	0.70 (0.03)	0.54 (0.03)	22.03 (2.52)

Data are mean (SD). VL10%: training until 10% of velocity loss (n = 8), VL20%: training until 20% of velocity loss (n = 7). MPV: Mean Propulsive Velocity; Fastest MPV: Average of the fastest repetitions measured in each session. Slowest MPV: Average of the slowest repetitions measured in each session.; Mean VL: Average velocity loss attained during the entire training program.

* p < 0.001 between groups

Table 2 shows the results and effect size for both groups before and after the 8-week intervention.

**Table 2:** Changes in selected athletic performance variables from pre-to post training for each group.

	VL10%		Effect Size (d)	VL20%		Effect Size (d)
	Pre	Post		Pre	Post	
1RM (kg) †	85.0 (12.5)	88.8 (13.0)*	0.29	71.43 (8.02)	76.43 (8.02)*	0.62
SJ (cm) ‡	28.7 (17.0)	31.3 (2.20)*	1.30	25.13 (3.84)	26.83 (4.42)	0.41
CMJ (cm) ‡	31.5 (3.90)	33.6 (1.80)	0.69	27.20 (4.50)	29.9 (4.60)	0.59
SP5 (m/s) ‡	4.46 (0.25)	4.50 (0.11)	0.18	4.24 (0.13)	4.28 (0.13)	0.34
SP10 (m/s) †	5.16 (0.19)	5.27 (0.14)*	0.62	4.96 (0.19)	5.03 (0.17)*	0.42
SP20 (m/s) †	5.81 (0.18)	5.92 (0.14)*	0.67	5.62 (0.18)	5.71 (0.16)*	0.56

Data are mean (SD). VL10%: training until 10% of velocity loss (n = 8), VL20%: training until 20% of velocity loss (n = 7).

1RM: 1 Repetition Maximum, SJ: Squat Jump, CMJ: Counter Movement Jump, SP5: 5m Sprint velocity, SP10: 10m Sprint velocity, SP20: 20m Sprint velocity. †: Parametrically analyzed, ‡: Nonparametric and analyzed. * p < 0.05 vs. Pre.

No significant between-group differences were observed in the change scores (Δ = post − pre) for SJ and CMJ (SJ: p = 0.288; CMJ: p = 0.216). Likewise, no significant between-group difference was observed for SP5 (p = 0.066). For parametric outcomes, two-way mixed ANOVA showed no significant between-group differences at pre-intervention for 1RM (p = 0.116), SP10 (p = 0.220), and SP20 (p = 0.232). Significant time effects were observed for 1RM (p = 0.008), SP10 (p = 0.004), and SP20 (p = 0.002); however, no significant group × time interactions were obtained. Additionally, the between-group difference in 1RM (Δ = post − pre) was not statistically significant (p = 0.554).

## Discussion

This study investigated the effects of velocity-based resistance training (VBT) with two different velocity loss (VL) thresholds—10% and 20%—on performance outcomes in trained female basketball players. Before interpreting the present findings, an important limitation should be acknowledged. Although the initial recruitment target approached the sample size required under standard assumptions, injury-related attrition reduced the final analytical sample. Consequently, the findings should be interpreted as estimates rather than confirmatory evidence, and the possibility of false-positive results cannot be excluded. Future studies with adequately powered sample sizes are required to robustly test group × time effects and to determine whether meaningful differences exist between velocity-loss thresholds.

Although previous studies conducted in male participants with larger sample sizes have reported similar improvements in strength, jump, and sprint performance following low and moderate velocity loss thresholds (e.g., VL5% vs. VL20%) [10], the present findings should be interpreted in light of important differences in participant characteristics. Unlike those studies, the present investigation focused on trained female basketball players, a population known to exhibit distinct neuromuscular and fatigue-related characteristics compared with males. Female athletes generally demonstrate greater fatigue resistance and a higher proportion of type I muscle fibers, which may alter the adaptive response to different velocity loss thresholds. Therefore, it is plausible that the optimal magnitude of velocity loss for maximizing performance adaptations differs between sexes and athletic populations. Moreover, differences in training status, sport-specific demands, and sample size may further contribute to the observed discrepancies between studies.

Despite the limited sample size, within-group results indicated improvements in SJ and sprint outcomes in the VL10% group, whereas changes in SJ were not clear within the VL20% group. However, between-group differences were not statistically significant, and the lack of a significant group × time interaction necessitates caution in interpreting any apparent differences between conditions. The present findings should be interpreted in the context of recent VBT literature, which has increasingly focused on the role of velocity loss thresholds in balancing training volume, fatigue, and performance adaptations intervention [3,4]. Recent evidence indicates that the magnitude of velocity loss during resistance training is strongly linked to task-specific adaptations rather than generalized improvements across all performance tests [4,15]. Our findings should also be interpreted in relation to the study by Rissanen et al. [15], who examined 20% versus 40% velocity-loss protocols in men and women. They reported overall improvements in strength and power-related outcomes in both sexes and suggested that women may benefit from a greater velocity loss, particularly for bench press adaptations, although statistical between-group differences were not observed. In contrast, the present study compared lower velocity-loss thresholds (10% vs 20%) and focused on sport-specific outcomes (jump and sprint performance) in trained female basketball players. Differences in the investigated velocity-loss range, participant characteristics, and outcome measures may explain why the practical implications of velocity-loss selection may not fully align across studies. Taken together, the available evidence suggests that the optimal velocity-loss threshold in women may depend on the targeted adaptation and the exercise selection (e.g., strength/upper-body outcomes vs. sprint- and jump-related performance).

### 1RM test

In the 1RM test, the group with a higher number of lifts (VL20%) shows a greater effect size after the training intervention, which is consistent with several previous studies [10,12,21]. Pareja-Blanco et al. [12] conducted an 8-week squat training program on 64 men with velocity loss (VL) of 0%, 10%, 20%, and 40%. The results showed that all groups improved their 1RM, with the greatest gains in the VL10% and VL20% groups in particular. This suggested that only one repetition per set (VL 0%) was insufficient to maximize strength gains, and that higher velocity losses (VL 40%) would not result in further gains. Furthermore, Pareja-Blanco et al. [21] compared VL20% and VL40% and reported greater quadriceps muscle hypertrophy at VL40%, but 1RM gains were comparable in both groups. In addition, there was a decrease in type IIX muscle fibers in the VL40% group, suggesting a negative effect on explosive muscle performance.

Based on the results of these studies, it can be inferred that the VL20% setting produced hypertrophy of the muscle fibers and an explosive improvement in muscle performance in this study and was one of the factors that significantly improved 1RM. The results of this study (with women) and previous studies (with men, [12,21]) also suggest that it is not always necessary to train to the limit of non-lifting to improve 1RM.

### Jump performance tests

In SJ, the movement does not involve an eccentric phase, but rather the movement begins with an explosive exertion of force by the lower extremity muscles [28]. The observed improvement in 1RM in the present study suggests that the improvement in lower limb muscle strength may have contributed to the improvement in SJ performance. However, The difference in the number of repetitions (training volume) may have influenced fatigue accumulation, such that the lower volume in VL10% may have resulted in less fatigue and thereby favored SJ performance despite relatively small 1RM improvements [10]. It has been shown that continued training at reduced training velocity (<0.6 m/s) may result in less adaptation to rapid force exertion due to a decrease in type IIX muscle fibers [21]. In the present study, VL20% trained at velocity below 0.6 m/s, while VL10% completed training at faster velocity (> 0.6 m/s). This may have resulted in a significant improvement in SJ for VL10% only. McBride, et al [28] also reported significant differences in concentric phase peak force, peak velocity, and jump height in CMJ compared to SJ. The results suggest that the presence or absence of an eccentric phase in the jumping motion may have an effect on performance. Eccentric-phase neuromuscular function is important in CMJ, and a possible reason for the lack of significant improvement in CMJ in this study may be a hypothesized lack of improvement in eccentric-phase adaptations [29,30].

### Sprint performance tests

A review by Howard et al. [31] reports that muscle activation in the hamstrings and quadriceps is active during the stance phase and increases with increasing sprint distance. It has also been reported that muscle activation in the rectus femoris muscle increases with increasing sprint speed [31]. Based on these findings, it is possible that high-velocity lift (> 0.6 m/s) training may have promoted neuromuscular factors relevant to sprint performance [8], which could have contributed to the improvements in SP10 and SP20 in the VL10% group [6]. On the other hand, Pareja-Blanco [12] confirmed that the rate of force rise at 50 ms (Rate of Force Development; RFD) was significantly reduced in the training group at VL40%. This study suggests that it may have been difficult to achieve the same amount of effect in the VL20% group, which had a higher number of lifts at relatively low velocity (< 0.6 m/s), as in the VL10% group. Styles WJ et al. [32] also reported that professional soccer players performing 4 sets of 5 squats at 85–90% 1RM showed significant performance gains at 5m, 10m, and 20m sprint distances, with the highest effect size at the 5m point. The loading condition in this study was 70% 1RM, which is less demanding than in previous study [32]. Therefore, it is possible that the present study did not adequately promote the adaptation of explosive muscular exertion. Furthermore, as suggested by the results of the present study, the combination of velocity training may be necessary to improve sprint performance over 10 m. In addition, future studies on the effects of VBT using high loads of around 85–90% 1RM in women, not only on sprint performance but also on other physical adaptations, are warranted. The effect size estimates reported in the present pilot study may also inform sample size planning for future confirmatory trials.

## Conclusions

This pilot study suggests that VBT using a 10% velocity loss threshold may improve squat jump performance and sprint split outcomes in trained female athletes, while both VL conditions showed comparable improvements in maximal strength. However, between-group differences were not statistically significant, and these results should be interpreted cautiously due to the small sample size. These findings suggest the potential utility of individualized VL settings to optimize training outcomes based on specific performance objectives.

However, due to the small sample size and high dropout rate, these results should be interpreted with caution. Further research involving larger, more diverse populations is needed to validate these findings and refine VBT prescriptions for female athletes.

## Supporting information

S1 File Data (Supplementary Information). (XLSX)
